# A monoclinic polymorph with *Z* = 4 of (*E*)-2,4-dihy­droxy­acetophenone 2,4-dinitro­phenyl­hydrazone *N*,*N*-dimethyl­formamide monosolvate

**DOI:** 10.1107/S1600536811050161

**Published:** 2011-11-25

**Authors:** Hongfei Han, Yaohua Liu

**Affiliations:** a Department of Chemistry, Taiyuan Normal University, Taiyuan 030031, People’s Republic of China

## Abstract

The title compound, C_14_H_12_N_4_O_6_·C_3_H_7_NO, is a monoclinic polymorph of an already published structure [Baughman *et al.* (2004 ▶). *Acta Cryst.* C**60**, 103–106]. In the previously reported structure, the compound crystallized in the triclinic space group *P* 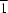 (*Z* = 2), whereas the structure reported here is monoclinic (*P*2_1_ */n*, *Z* = 4). In both forms, two intra­molecular hydrogen bonds result in the formation of a fairly planar hydrazone skeleton (r.m.s. deviations for all non-H atoms = 0.127 Å for the monoclinic from and 0.131 Å for the triclinic form) and each mol­ecule is hydrogen bonded to one solvent mol­ecule. The principal difference between the two forms lies in the different orientation of the two mol­ecules. In the monoclinic form, the two mol­ecules are almost coplanar [dihedral angle = 3.27 (2)°], whereas in the triclinic form the two mol­ecules are almost mutulally perpendicular (dihedral angle = 85.3°).

## Related literature

For the biological activity of Schiff bases, see: Khan *et al.* (2009 ▶); Gerdemann *et al.* (2002 ▶); Mallikarjun & Sangamesh (1997 ▶); Solomon & Lowery (1993 ▶). For the crystal structure of the triclinic polymorph, see: Baughman *et al.* (2004 ▶).
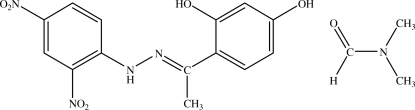


## Experimental

### 

#### Crystal data

C_14_H_12_N_4_O_6_·C_3_H_7_NO
*M* *_r_* = 405.37
Monoclinic, 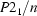

*a* = 6.7546 (6) Å
*b* = 20.9647 (18) Å
*c* = 13.3508 (13) Å
β = 99.772 (1)°
*V* = 1863.2 (3) Å^3^
*Z* = 4
Mo *K*α radiation
μ = 0.11 mm^−1^
*T* = 298 K
0.43 × 0.28 × 0.24 mm

#### Data collection

Bruker SMART CCD area-detector diffractometer
Absorption correction: multi-scan (*SADABS*; Sheldrick, 1996 ▶) *T*_min_ = 0.953, *T*_max_ = 0.973
9407 measured reflections
3280 independent reflections
1642 reflections with *I* > 2σ(*I*)
*R*_int_ = 0.058

#### Refinement

*R*[*F* ^2^ > 2σ(*F* ^2^)] = 0.052
*wR*(*F* ^2^) = 0.142
*S* = 0.88
3280 reflections
262 parameters
H-atom parameters constrained
Δρ_max_ = 0.25 e Å^−3^
Δρ_min_ = −0.23 e Å^−3^

### 

Data collection: *SMART* (Bruker, 2007 ▶); cell refinement: *SAINT* (Bruker, 2007 ▶); data reduction: *SAINT*; program(s) used to solve structure: *SHELXS97* (Sheldrick, 2008 ▶); program(s) used to refine structure: *SHELXL97* (Sheldrick, 2008 ▶); molecular graphics: *SHELXTL* (Sheldrick, 2008 ▶); software used to prepare material for publication: *SHELXTL*.

## Supplementary Material

Crystal structure: contains datablock(s) I, global. DOI: 10.1107/S1600536811050161/bt5693sup1.cif

Structure factors: contains datablock(s) I. DOI: 10.1107/S1600536811050161/bt5693Isup2.hkl

Supplementary material file. DOI: 10.1107/S1600536811050161/bt5693Isup3.cml

Additional supplementary materials:  crystallographic information; 3D view; checkCIF report

## Figures and Tables

**Table 1:** Hydrogen-bond geometry (Å, °)

*D*—H⋯*A*	*D*—H	H⋯*A*	*D*⋯*A*	*D*—H⋯*A*
O1—H1⋯N1	0.82	1.82	2.547 (2)	146
O2—H2*A*⋯O7	0.82	1.81	2.611 (3)	164
N2—H2⋯O3	0.86	1.94	2.584 (3)	130

**Fig. 1.**
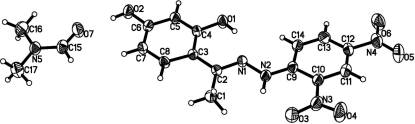
The molecular structure, showing the atom-numbering scheme. Displacement ellipsoids are drawn at the 30% probability level. H atoms have been omitted for clarity.

## supplementary crystallographic information

### Comment

The Schiff bases containing the C═N bond have been receiving considerable attention for many years, primarily due to a wide range of biological properties including antifungal, antibacterial, herbicidal, antiproliferative, cytotoxic, anticonvulsant and anticancer activities (Khan *et al.*, 2009; Gerdemann *et al.*, 2002; Mallikarjun & Sangamesh, 1997; Solomon & Lowery, 1993). The title compound, (I), is a monoclinic polymorph of the previously reported crystal structure which crystallizes in the triclinic space group *P*1 (Baughman *et al.*, 2004). The relative arrangement of the molecules observed in the current structure is different from that previously reported.

The molecular structure of (I) is shown in Fig. 1. It crystallizes in the space group *P*2_1_*/n*, with four molecules in each unit cell. The azomethine double bond adopts an E configuration. The solvent molecule and Schiff base molecule are linked by O—H···N hydrogen bond. The planes of the solvent molecule and adjacent benzene ring linked by hydrogen bond are almost parallel. The dihedral angle is 0.209 (127). One N—H···O, one O—H···N and one O—H···O hydrogen bonds link the molecules, forming a two-dimensional network. Whereas in (II) the dihedral angle between the planes of the solvent molecule and adjacent benzene ring linked by O—H···N hydrogen bond is 86.619 (143). Besides above three kind of hydrogen bonds, intermolecular O5···O5^i^ interaction (symmetry code i: 1 - *x*, 1 - *y*, 1 - *z*) link the molecules into a three-dimensional network.

### Experimental

The synthesis of title compound I was carried out by refluxing a mixture of 2,4-dihydroxyacetophenone (0.76 g, 5 mmol) and 2,4-dinitrophenylhydrazine (0.99 g, 5 mmol) with concentrated sulfuric acid (5 mL) in ethanol (20 mL) for 2 h. After cooling and filtration the crystalline product was collected, washed with hexane and dried to afford the title compound in 85% yield.

### Refinement

All H atoms were positioned geometrically (C—H = 0.93–0.96 Å, O—H 0.82Å, N—H = 0.86Å), and refined as riding with *U*_iso_(H) = 1.2*U*_eq_ or 1.5*U*_eq_ (methyl H atoms).

### Crystal data

**Table:**

C_14_H_12_N_4_O_6_·C_3_H_7_NO	*F*(000) = 848
*M**_r_* = 405.37	*D*_x_ = 1.445 Mg m^−^^3^
Monoclinic, *P*2_1_/*n*	Mo *K*α radiation, λ = 0.71073 Å
Hall symbol: -P 2yn	Cell parameters from 1386 reflections
*a* = 6.7546 (6) Å	θ = 3.0–21.9°
*b* = 20.9647 (18) Å	µ = 0.11 mm^−^^1^
*c* = 13.3508 (13) Å	*T* = 298 K
β = 99.772 (1)°	Block, brown
*V* = 1863.2 (3) Å^3^	0.43 × 0.28 × 0.24 mm
*Z* = 4	

### Data collection

**Table:**

Bruker SMART CCD area-detector diffractometer	3280 independent reflections
Radiation source: fine-focus sealed tube	1642 reflections with *I* > 2σ(*I*)
graphite	*R*_int_ = 0.058
phi and ω scans	θ_max_ = 25.0°, θ_min_ = 2.5°
Absorption correction: multi-scan (*SADABS*; Sheldrick, 1996)	*h* = −8→7
*T*_min_ = 0.953, *T*_max_ = 0.973	*k* = −17→24
9407 measured reflections	*l* = −15→15

### Refinement

**Table:**

Refinement on *F*^2^	Primary atom site location: structure-invariant direct methods
Least-squares matrix: full	Secondary atom site location: difference Fourier map
*R*[*F*^2^ > 2σ(*F*^2^)] = 0.052	Hydrogen site location: inferred from neighbouring sites
*wR*(*F*^2^) = 0.142	H-atom parameters constrained
*S* = 0.88	*w* = 1/[σ^2^(*F*_o_^2^) + (0.0687*P*)^2^] where *P* = (*F*_o_^2^ + 2*F*_c_^2^)/3
3280 reflections	(Δ/σ)_max_ < 0.001
262 parameters	Δρ_max_ = 0.25 e Å^−^^3^
0 restraints	Δρ_min_ = −0.23 e Å^−^^3^

### Special details

**Table:**

Geometry. All e.s.d.'s (except the e.s.d. in the dihedral angle between two l.s. planes) are estimated using the full covariance matrix. The cell e.s.d.'s are taken into account individually in the estimation of e.s.d.'s in distances, angles and torsion angles; correlations between e.s.d.'s in cell parameters are only used when they are defined by crystal symmetry. An approximate (isotropic) treatment of cell e.s.d.'s is used for estimating e.s.d.'s involving l.s. planes.
Refinement. Refinement of *F*^2^ against ALL reflections. The weighted *R*-factor *wR* and goodness of fit *S* are based on *F*^2^, conventional *R*-factors *R* are based on *F*, with *F* set to zero for negative *F*^2^. The threshold expression of *F*^2^ > σ(*F*^2^) is used only for calculating *R*-factors(gt) *etc*. and is not relevant to the choice of reflections for refinement. *R*-factors based on *F*^2^ are statistically about twice as large as those based on *F*, and *R*- factors based on ALL data will be even larger.

### Fractional atomic coordinates and isotropic or equivalent isotropic displacement parameters (Å^2^)

**Table:**

	*x*	*y*	*z*	*U*_iso_*/*U*_eq_	
N1	0.7588 (3)	0.58432 (9)	0.54589 (14)	0.0446 (5)	
N2	0.7718 (3)	0.63470 (9)	0.61267 (15)	0.0479 (6)	
H2	0.7949	0.6272	0.6769	0.058*	
N3	0.7472 (4)	0.73692 (13)	0.75436 (18)	0.0709 (7)	
N4	0.6738 (4)	0.88617 (12)	0.4785 (2)	0.0705 (7)	
N5	0.8095 (4)	0.11311 (11)	0.45642 (17)	0.0620 (7)	
O1	0.6979 (3)	0.54234 (8)	0.36429 (12)	0.0644 (6)	
H1	0.7114	0.5692	0.4096	0.097*	
O2	0.7018 (3)	0.32319 (8)	0.30681 (14)	0.0683 (6)	
H2A	0.7141	0.2888	0.3365	0.102*	
O3	0.7879 (4)	0.68408 (10)	0.79108 (14)	0.0790 (7)	
O4	0.7151 (5)	0.78168 (11)	0.80637 (17)	0.1192 (11)	
O5	0.6748 (4)	0.92791 (10)	0.5426 (2)	0.0961 (8)	
O6	0.6526 (4)	0.89703 (10)	0.3875 (2)	0.0934 (8)	
O7	0.7736 (4)	0.20700 (9)	0.37332 (18)	0.0801 (7)	
C1	0.8138 (4)	0.51378 (13)	0.69559 (18)	0.0575 (7)	
H1A	0.9127	0.5430	0.7293	0.086*	
H1B	0.8615	0.4708	0.7074	0.086*	
H1C	0.6909	0.5190	0.7217	0.086*	
C2	0.7768 (4)	0.52710 (12)	0.58373 (18)	0.0422 (6)	
C3	0.7571 (4)	0.47439 (11)	0.51209 (18)	0.0409 (6)	
C4	0.7195 (4)	0.48320 (11)	0.40614 (19)	0.0454 (7)	
C5	0.7022 (4)	0.43264 (12)	0.34011 (19)	0.0507 (7)	
H5	0.6773	0.4401	0.2704	0.061*	
C6	0.7211 (4)	0.37116 (12)	0.37582 (19)	0.0491 (7)	
C7	0.7566 (4)	0.36059 (11)	0.47885 (19)	0.0507 (7)	
H7	0.7688	0.3191	0.5039	0.061*	
C8	0.7739 (4)	0.41082 (12)	0.54402 (19)	0.0488 (7)	
H8	0.7982	0.4025	0.6134	0.059*	
C9	0.7493 (4)	0.69475 (11)	0.58042 (19)	0.0448 (7)	
C10	0.7384 (4)	0.74617 (12)	0.64741 (19)	0.0500 (7)	
C11	0.7124 (4)	0.80809 (12)	0.6129 (2)	0.0557 (8)	
H11	0.7038	0.8411	0.6585	0.067*	
C12	0.6994 (4)	0.82073 (12)	0.5123 (2)	0.0540 (7)	
C13	0.7114 (4)	0.77217 (13)	0.4441 (2)	0.0576 (8)	
H13	0.7031	0.7814	0.3753	0.069*	
C14	0.7354 (4)	0.71070 (12)	0.4767 (2)	0.0531 (7)	
H14	0.7429	0.6785	0.4297	0.064*	
C15	0.8017 (4)	0.17571 (14)	0.4520 (3)	0.0641 (8)	
H15	0.8188	0.1979	0.5132	0.077*	
C16	0.7826 (6)	0.07595 (15)	0.3644 (2)	0.0991 (12)	
H16A	0.7287	0.1026	0.3078	0.149*	
H16B	0.6913	0.0415	0.3697	0.149*	
H16C	0.9097	0.0590	0.3543	0.149*	
C17	0.8457 (5)	0.07943 (14)	0.5520 (2)	0.0817 (10)	
H17A	0.8718	0.1096	0.6067	0.123*	
H17B	0.9598	0.0519	0.5539	0.123*	
H17C	0.7297	0.0544	0.5588	0.123*	

### Atomic displacement parameters (Å^2^)

**Table:**

	*U*^11^	*U*^22^	*U*^33^	*U*^12^	*U*^13^	*U*^23^
N1	0.0514 (14)	0.0378 (12)	0.0452 (12)	0.0018 (10)	0.0102 (11)	0.0009 (10)
N2	0.0622 (16)	0.0410 (13)	0.0408 (12)	0.0006 (11)	0.0091 (11)	−0.0022 (10)
N3	0.105 (2)	0.0534 (16)	0.0547 (16)	−0.0029 (15)	0.0131 (15)	−0.0086 (14)
N4	0.0691 (19)	0.0478 (16)	0.096 (2)	0.0019 (13)	0.0174 (17)	0.0128 (16)
N5	0.0782 (19)	0.0438 (14)	0.0644 (16)	0.0041 (13)	0.0128 (14)	−0.0072 (12)
O1	0.1061 (17)	0.0411 (11)	0.0449 (11)	0.0005 (10)	0.0097 (11)	0.0061 (9)
O2	0.1046 (17)	0.0450 (11)	0.0560 (12)	−0.0050 (11)	0.0153 (11)	−0.0062 (9)
O3	0.124 (2)	0.0610 (14)	0.0511 (13)	0.0039 (13)	0.0134 (12)	0.0014 (10)
O4	0.229 (3)	0.0657 (15)	0.0651 (16)	0.0233 (17)	0.0323 (18)	−0.0183 (12)
O5	0.126 (2)	0.0439 (12)	0.121 (2)	0.0026 (13)	0.0292 (17)	−0.0012 (13)
O6	0.124 (2)	0.0651 (15)	0.0915 (17)	0.0042 (13)	0.0190 (16)	0.0273 (14)
O7	0.0996 (19)	0.0539 (13)	0.0869 (17)	0.0088 (12)	0.0155 (14)	0.0077 (12)
C1	0.073 (2)	0.0522 (16)	0.0456 (16)	0.0012 (15)	0.0062 (14)	0.0007 (13)
C2	0.0428 (17)	0.0412 (15)	0.0430 (15)	0.0024 (12)	0.0088 (12)	0.0017 (12)
C3	0.0412 (16)	0.0381 (14)	0.0440 (15)	0.0007 (12)	0.0091 (12)	0.0033 (12)
C4	0.0507 (18)	0.0378 (15)	0.0485 (16)	−0.0007 (13)	0.0105 (13)	0.0052 (13)
C5	0.064 (2)	0.0439 (16)	0.0432 (16)	−0.0016 (14)	0.0078 (14)	0.0007 (13)
C6	0.0557 (19)	0.0429 (16)	0.0496 (17)	−0.0043 (13)	0.0115 (14)	−0.0054 (13)
C7	0.0612 (19)	0.0375 (15)	0.0540 (17)	0.0019 (13)	0.0119 (14)	0.0046 (13)
C8	0.0597 (19)	0.0451 (16)	0.0424 (15)	0.0030 (13)	0.0112 (13)	0.0067 (13)
C9	0.0456 (17)	0.0384 (15)	0.0509 (16)	−0.0031 (12)	0.0092 (13)	0.0007 (13)
C10	0.0584 (19)	0.0451 (16)	0.0466 (16)	−0.0010 (13)	0.0088 (13)	−0.0037 (13)
C11	0.059 (2)	0.0449 (17)	0.0644 (19)	−0.0024 (14)	0.0144 (15)	−0.0097 (14)
C12	0.0520 (19)	0.0393 (16)	0.071 (2)	0.0034 (13)	0.0125 (15)	0.0059 (14)
C13	0.064 (2)	0.0564 (19)	0.0527 (17)	−0.0011 (15)	0.0102 (15)	0.0083 (14)
C14	0.065 (2)	0.0456 (16)	0.0485 (17)	−0.0003 (14)	0.0101 (14)	−0.0017 (13)
C15	0.064 (2)	0.050 (2)	0.080 (2)	0.0019 (16)	0.0179 (18)	−0.0065 (17)
C16	0.143 (4)	0.067 (2)	0.080 (2)	0.010 (2)	0.001 (2)	−0.0239 (19)
C17	0.097 (3)	0.065 (2)	0.086 (2)	0.0105 (19)	0.020 (2)	0.0072 (18)

### Geometric parameters (Å, °)

**Table:**

N1—C2	1.299 (3)	C3—C4	1.406 (3)
N1—N2	1.375 (2)	C4—C5	1.371 (3)
N2—C9	1.331 (3)	C5—C6	1.373 (3)
N2—H2	0.8600	C5—H5	0.9300
N3—O4	1.209 (3)	C6—C7	1.374 (3)
N3—O3	1.224 (3)	C7—C8	1.358 (3)
N3—C10	1.432 (3)	C7—H7	0.9300
N4—O6	1.220 (3)	C8—H8	0.9300
N4—O5	1.223 (3)	C9—C10	1.411 (3)
N4—C12	1.445 (3)	C9—C14	1.412 (3)
N5—C15	1.314 (3)	C10—C11	1.379 (3)
N5—C17	1.442 (3)	C11—C12	1.357 (4)
N5—C16	1.440 (3)	C11—H11	0.9300
O1—C4	1.358 (3)	C12—C13	1.378 (4)
O1—H1	0.8200	C13—C14	1.361 (3)
O2—C6	1.355 (3)	C13—H13	0.9300
O2—H2A	0.8200	C14—H14	0.9300
O7—C15	1.226 (3)	C15—H15	0.9300
C1—C2	1.498 (3)	C16—H16A	0.9600
C1—H1A	0.9600	C16—H16B	0.9600
C1—H1B	0.9600	C16—H16C	0.9600
C1—H1C	0.9600	C17—H17A	0.9600
C2—C3	1.453 (3)	C17—H17B	0.9600
C3—C8	1.398 (3)	C17—H17C	0.9600
			
C2—N1—N2	117.74 (19)	C6—C7—H7	120.1
C9—N2—N1	121.7 (2)	C7—C8—C3	123.4 (2)
C9—N2—H2	119.1	C7—C8—H8	118.3
N1—N2—H2	119.1	C3—C8—H8	118.3
O4—N3—O3	121.5 (2)	N2—C9—C10	122.2 (2)
O4—N3—C10	119.1 (3)	N2—C9—C14	121.8 (2)
O3—N3—C10	119.4 (2)	C10—C9—C14	116.0 (2)
O6—N4—O5	123.3 (3)	C11—C10—C9	121.7 (2)
O6—N4—C12	118.4 (3)	C11—C10—N3	116.2 (2)
O5—N4—C12	118.4 (3)	C9—C10—N3	122.1 (2)
C15—N5—C17	121.9 (3)	C12—C11—C10	119.8 (3)
C15—N5—C16	120.3 (3)	C12—C11—H11	120.1
C17—N5—C16	117.9 (2)	C10—C11—H11	120.1
C4—O1—H1	109.5	C11—C12—C13	120.6 (2)
C6—O2—H2A	109.5	C11—C12—N4	118.6 (3)
C2—C1—H1A	109.5	C13—C12—N4	120.8 (3)
C2—C1—H1B	109.5	C14—C13—C12	120.3 (3)
H1A—C1—H1B	109.5	C14—C13—H13	119.8
C2—C1—H1C	109.5	C12—C13—H13	119.8
H1A—C1—H1C	109.5	C13—C14—C9	121.5 (2)
H1B—C1—H1C	109.5	C13—C14—H14	119.2
N1—C2—C3	117.0 (2)	C9—C14—H14	119.2
N1—C2—C1	123.3 (2)	O7—C15—N5	124.9 (3)
C3—C2—C1	119.7 (2)	O7—C15—H15	117.6
C8—C3—C4	115.0 (2)	N5—C15—H15	117.6
C8—C3—C2	122.1 (2)	N5—C16—H16A	109.5
C4—C3—C2	122.9 (2)	N5—C16—H16B	109.5
O1—C4—C5	116.7 (2)	H16A—C16—H16B	109.5
O1—C4—C3	121.5 (2)	N5—C16—H16C	109.5
C5—C4—C3	121.8 (2)	H16A—C16—H16C	109.5
C4—C5—C6	120.7 (2)	H16B—C16—H16C	109.5
C4—C5—H5	119.7	N5—C17—H17A	109.5
C6—C5—H5	119.7	N5—C17—H17B	109.5
O2—C6—C5	117.9 (2)	H17A—C17—H17B	109.5
O2—C6—C7	122.8 (2)	N5—C17—H17C	109.5
C5—C6—C7	119.3 (2)	H17A—C17—H17C	109.5
C8—C7—C6	119.9 (2)	H17B—C17—H17C	109.5
C8—C7—H7	120.1		
			
C2—N1—N2—C9	−178.1 (2)	C14—C9—C10—C11	1.0 (4)
N2—N1—C2—C3	178.3 (2)	N2—C9—C10—N3	−1.1 (4)
N2—N1—C2—C1	−1.0 (4)	C14—C9—C10—N3	179.0 (3)
N1—C2—C3—C8	179.9 (2)	O4—N3—C10—C11	6.1 (4)
C1—C2—C3—C8	−0.8 (4)	O3—N3—C10—C11	−173.5 (3)
N1—C2—C3—C4	−0.2 (4)	O4—N3—C10—C9	−172.0 (3)
C1—C2—C3—C4	179.1 (2)	O3—N3—C10—C9	8.4 (4)
C8—C3—C4—O1	179.4 (2)	C9—C10—C11—C12	−0.9 (4)
C2—C3—C4—O1	−0.5 (4)	N3—C10—C11—C12	−179.0 (3)
C8—C3—C4—C5	−0.3 (4)	C10—C11—C12—C13	0.2 (4)
C2—C3—C4—C5	179.8 (2)	C10—C11—C12—N4	−179.5 (3)
O1—C4—C5—C6	−179.8 (2)	O6—N4—C12—C11	−176.6 (3)
C3—C4—C5—C6	0.0 (4)	O5—N4—C12—C11	3.5 (4)
C4—C5—C6—O2	179.8 (2)	O6—N4—C12—C13	3.8 (4)
C4—C5—C6—C7	0.4 (4)	O5—N4—C12—C13	−176.1 (3)
O2—C6—C7—C8	−179.8 (2)	C11—C12—C13—C14	0.4 (4)
C5—C6—C7—C8	−0.5 (4)	N4—C12—C13—C14	−180.0 (3)
C6—C7—C8—C3	0.1 (4)	C12—C13—C14—C9	−0.2 (4)
C4—C3—C8—C7	0.3 (4)	N2—C9—C14—C13	179.6 (2)
C2—C3—C8—C7	−179.8 (2)	C10—C9—C14—C13	−0.5 (4)
N1—N2—C9—C10	172.3 (2)	C17—N5—C15—O7	179.2 (3)
N1—N2—C9—C14	−7.8 (4)	C16—N5—C15—O7	−0.5 (5)
N2—C9—C10—C11	−179.1 (3)		

### Hydrogen-bond geometry (Å, °)

**Table:**

*D*—H···*A*	*D*—H	H···*A*	*D*···*A*	*D*—H···*A*
O1—H1···N1	0.82	1.82	2.547 (2)	146
O2—H2A···O7	0.82	1.81	2.611 (3)	164
N2—H2···O3	0.86	1.94	2.584 (3)	130
